# Adjuvant Chemotherapy Benefit in Elderly Stage II/III Colon Cancer Patients

**DOI:** 10.3389/fonc.2022.874749

**Published:** 2022-06-07

**Authors:** Xin Chen, Junhao Tu, Xiaolan Xu, Wen Gu, Lei Qin, Haixin Qian, Zhenyu Jia, Chuntao Ma, Yinkai Xu

**Affiliations:** ^1^ Department of General Surgery, The First Affiliated Hospital of Soochow University, Suzhou, China; ^2^ Department of General Surgery, Suzhou Wuzhong People’s Hospital, Suzhou, China; ^3^ Department of Gastroenterology, Xiangcheng People’s Hospital, Suzhou, China; ^4^ Department of Gastroenterology, The First Affiliated Hospital of Soochow University, Suzhou, China

**Keywords:** chemotherapy, survival, elderly, stage II/III, colon cancer

## Abstract

**Background:**

Studies providing more evidence to guide adjuvant chemotherapy decisions in elderly colon cancer patients are expected.

**Methods:**

We obtained data from the Surveillance, Epidemiology and End Results (SEER) database between 2004 and 2012. Kaplan-Meier survival curves were constructed to calculate the cancer-specific survival (CSS) rate, and comparisons of survival difference between different subgroups were performed using the log-rank test. Multivariate Cox proportional hazards regression models were carried out to estimate hazard ratio (HR) and 95% confidence intervals (CIs) of different clinicopathological characteristics.

**Results:**

In stage II colon cancer patients aged 70 years or older, the Kaplan-Meier survival analysis showed that the 5-year CSS rates of no chemotherapy and chemotherapy groups were 82.0% and 72.4%, respectively (P < 0.001). In stage III colon cancer patients aged 70 years or older, the Kaplan-Meier survival analysis showed that the 5-year CSS rates of no chemotherapy and chemotherapy groups were 50.7% and 61.3%, respectively (P < 0.001). Patients with chemotherapy receipt were independently associated with a 35.8% lower cancer-specific mortality rate (HR = 0.642, 95% CI: 0.620-0.665, P < 0.001) compared with those who did not receive chemotherapy.

**Conclusions:**

Adjuvant chemotherapy should be considered during the treatment of stage III colon cancer patients aged 70 years or older, but the chemotherapy benefit in elderly stage II colon cancer is suboptimal.

## Introduction

As an important public health issue, colorectal cancer (CRC) is the third most commonly diagnosed cancer and the fourth most common cause of cancer death around the world (1).

With the aging of the population, the elderly occupies a sizable proportion of colon cancer patients and 60% percent of them are diagnosed over the age of 65 years (2). Older patients with colon cancer are reported to have worse survival compared with younger patients (3–6).

In 2001, a well-known randomized controlled trial reported that 5-FU-based adjuvant therapy could improve patient outcomes without a significant increase in toxic effects in selected elderly patients with colon cancer (7). Nonetheless, elderly CRC patients have been underrepresented in clinical trials, which has been a long-standing issue and needs further attention (8). In clinical trials, patients who are aged 65 years or older account for only 40% of patients, compared to 72% of the US population. Moreover, patients who are aged 70 years or older account for only 14% in clinical trials (9).

It has been previously proposed that 5-FU-based adjuvant therapy has shown comparable toxicity rates and similar survival benefits in elderly colon cancer patients compared with younger patients (7, 10). However, these studies were conducted before the incorporation of oxaliplatin into the 5-FU-based adjuvant therapy. In 2004, the addition of oxaliplatin to 5-FU/leucovorin (LV) chemotherapy was shown to improve disease-free survival (DFS) rate by 24% and reduce mortality rate by 14% compared with 5-FU/LV therapy alone in the MOSAIC trial (11).

In 2013, a study in Korea evaluated the efficacy of adjuvant chemotherapy in elderly colon cancer patients, which showed the survival benefit in stage III colon cancer patients offered by adjuvant chemotherapy, but the number of patients included in their study was too small (n= 382) (12). Therefore, the efficacy of adjuvant therapy has been reported in only a highly selected group of elderly colon cancer patients and studies providing more evidence to guide adjuvant chemotherapy decisions in elderly colon cancer patients are expected.

## Methods

### Data Source

**Figure 1:**
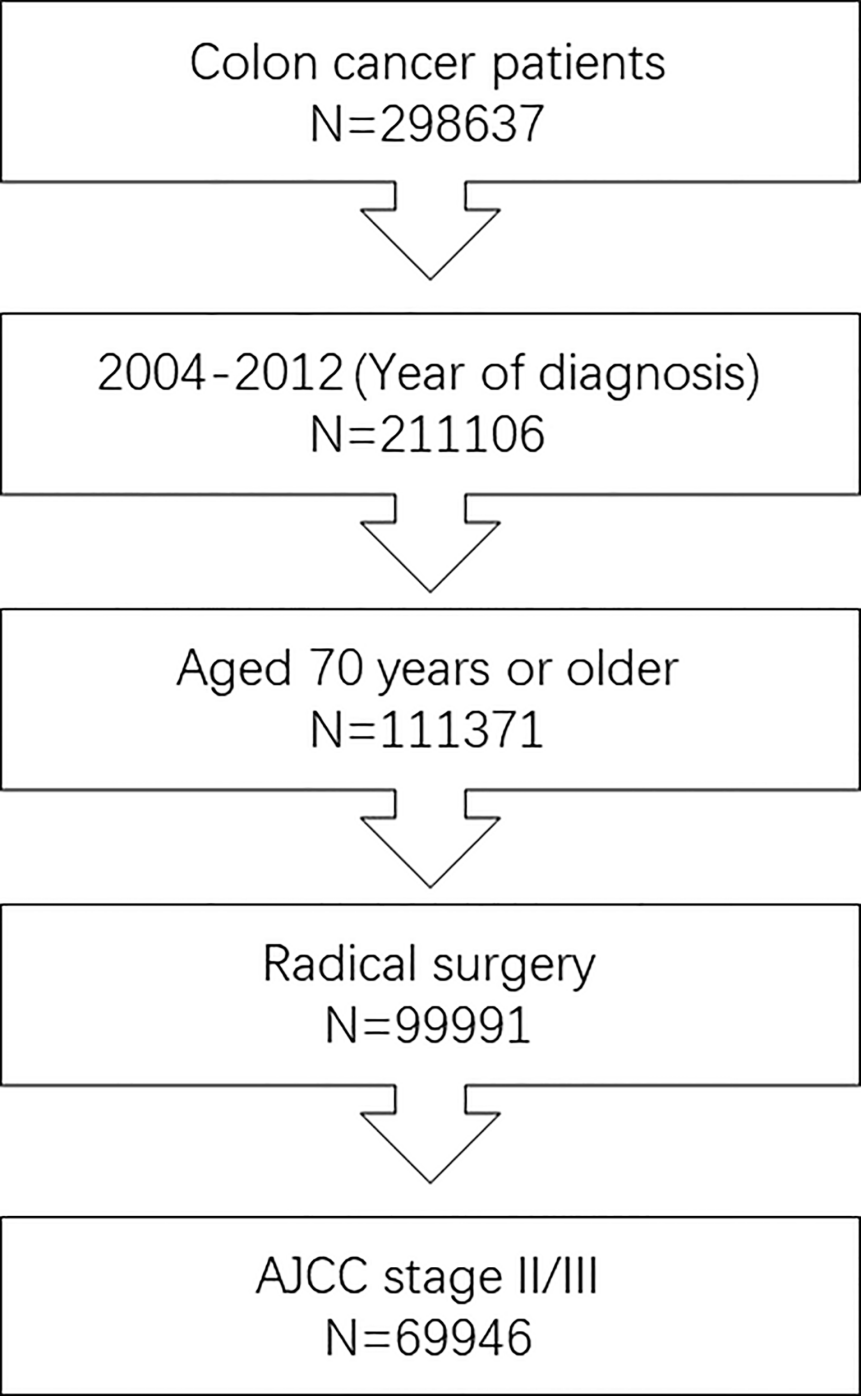
Flowchart of cohort ascertainment in this study. We included 69,946 stage II/III patients aged 70 years or older diagnosed with colon cancer between 2004 and 2012.

In this retrospective analysis, we obtained data from the Surveillance, Epidemiology and End Results (SEER) database between 2004 and 2012, which was maintained by the National Cancer Institute and covered basic information for ∼28% of the US population. A flowchart of cohort ascertainment is shown in **Figure 1**. The patient information of the SEER database is publicly available and the SEER database is free of any sensitive patient information or identifiers. Therefore, the collection of it did not require informed consent and the ethical approval was not required for this study. From the database, the following patient and tumor information was collected: survival status, survival months, TNM stage, race, sex, year of diagnosis, tumor grade, primary tumor site, histology, treatment, and patient age.

### Statistical Analysis

The cancer-specific survival (CSS) was the time from the initial colon cancer diagnosis to colon cancer-associated death. Only deaths caused by colon cancer were regarded as events, and deaths from other causes were censored at the date of death. Chi-square test was performed to compare categorical data. Kaplan-Meier survival curves were plotted to calculate the CSS rate, and comparisons of survival difference between different subgroups were performed using the log-rank test. Multivariate Cox proportional hazards regression models were conducted to estimate hazard ratio (HR) and 95% confidence intervals (CIs) of different clinicopathological characteristics as follows: TNM stage, race, sex, year of diagnosis, tumor grade, primary tumor site, histology, and chemotherapy. The statistical analyses were conducted by using the statistical software package SPSS 23.0 (IBM Corp, Armonk, NY). All statistical tests were two-sided and a P value less than 0.05 was regarded as statistically significant.

## Results

### Baseline Characteristics of Stage II/III Colon Cancer Patients

Finally, we included 69,946 stage II/III patients aged 70 years or older diagnosed with colon cancer between 2004 and 2012. In patients with known TNM stage, 35,099 (50.2%) patients were diagnosed with stage II colon cancer, which was comparable to stage III colon cancer patients; in colon cancer patients with known race, patients of white race (59,226, 84.7%) were far more prevalent than other races (10,720, 15.3%); in colon patients with known gender, male (31,024, 44.4%) patients were less prevalent than female (38,922, 55.6%) patients; in colon patients with known year of diagnosis, more patients were diagnosed with colon cancer between 2004 and 2008 (41,357, 59.1%) than between 2009 and 2012 (28,589, 40.9%); in colon patients with known tumor grade, grade I/II (50,234, 71.8%) patients were far more than grade III/IV (18,261, 26.1%) patients; in colon patients with known primary tumor site, patients with right colon cancer (48,640, 69.5%) were significantly more than patients with left colon cancer (21,306, 30.5%); in colon patients with known histology, patients with adenocarcinoma (61,708, 88.2%) were far more than patients with mucinous adenocarcinoma/signet ring cell carcinoma (8238, 11.8%).

**Table 1** summarized the demographic and clinicopathological characteristics of stage II/III colon cancer patients by the receipt of chemotherapy. Significant differences were found in TNM stage, race, sex, year of diagnosis, tumor grade, and primary tumor site by the receipt of chemotherapy. Stage III (81.7% vs. 39.8%), other races (17.0% vs. 14.8%), male (48.0% vs. 43.2%), year of diagnosis between 2009 and 2012 (42.0% vs. 40.5%), grade III/IV (30.7% vs. 24.7%), and left colon cancer (32.9% vs. 29.7%) were more likely to be associated with the receipt of adjuvant chemotherapy (P < 0.05).

**Table 1:** Clinicopathological characteristics of colon cancer patients by the receipt of chemotherapy.

Variables	Chemotherapy (%)		P value
	No (N=53,233)	Yes (N=16,713)	
**Stage**			<.001
**II**	32,046 (60.2)	3053 (18.3)	
**III**	21,187 (39.8)	13,660 (81.7)	
**Race**			<.001
**White**	45,349 (85.2)	13,877 (83.0)	
**Other**	7884 (14.8)	2836 (17.0)	
**Sex**			<.001
**Male**	22,995 (43.2)	8029 (48.0)	
**Female**	30,238 (56.8)	8684 (52.0)	
**Year of diagnosis**			.001
**2004-2008**	31,656 (59.5)	9701 (58.0)	
**2009-2012**	21,577 (40.5)	7012 (42.0)	
**Grade**			<.001
**I/II**	39,007 (73.3)	11,227 (67.2)	
**III/IV**	13,128 (24.7)	5133 (30.7)	
**Unknown**	1098 (2.1)	353 (2.1)	
**Primary tumor site**			<.001
**Right colon**	37,422 (70.3)	11,218 (67.1)	
**Left colon**	15,811 (29.7)	5495 (32.9)	
**Histology**			.385
**Adenocarcinoma**	46,995 (88.3)	14,713 (88.0)	
**Mucinous adenocarcinoma/signet ring cell carcinoma**	6238 (11.7)	2000 (12.0)	

**Table 2** summarizes the demographic and clinicopathological characteristics of colon cancer patients by different stages. Significant differences were found in the receipt of adjuvant chemotherapy, race, tumor grade, and histology. Compared with stage II patients, stage III colon cancer patients were more likely to be associated with the receipt of adjuvant chemotherapy (39.2% vs. 8.7%), other races (16.6% vs. 14.0%), grade III/IV (32.6% vs. 19.6%), and mucinous adenocarcinoma/signet ring cell carcinoma (12.1% vs. 11.5%) with two-sided P values less than 0.05.

**Table 2:** Clinicopathological characteristics of colon cancer patients by stage group.

Variables	(%)		P value
	II (N=35099)	III (N=34847)	
**Chemotherapy**			<.001
**No**	32,046 (91.3)	21,187 (60.8)	
**Yes**	3053 (8.7)	13,660 (39.2)	
**Race**			<.001
**White**	30,170 (86.0)	29,056 (83.4)	
**Other**	4929 (14.0)	5791 (16.6)	
**Sex**			.977
**Male**	15,566 (44.3)	15,458 (44.4)	
**Female**	19,533 (55.7)	19,389 (55.6)	
**Year of diagnosis**			.051
**2004-2008**	20,880 (59.5)	20,477 (58.8)	
**2009-2012**	14,219 (40.5)	14,370 (41.2)	
**Grade**			<.001
**I/II**	27,528 (78.4)	22,706 (65.2)	
**III/IV**	6886 (19.6)	11,375 (32.6)	
**Unknown**	685 (2.0)	766 (2.2)	
**Primary tumor site**			.874
**Right colon**	24,398 (69.5)	24,242 (69.6)	
**Left colon**	10,701 (30.5)	10,605 (30.4)	
**Histology**			.009
**Adenocarcinoma**	31,077 (88.5)	30,631 (87.9)	
**Mucinous adenocarcinoma/signet ring cell carcinoma**	4022 (11.5)	4216 (12.1)	

### The Effect of Adjuvant Chemotherapy in Stage II Colon Cancer Patients Aged 70 Years or Older

**Figure 2:**
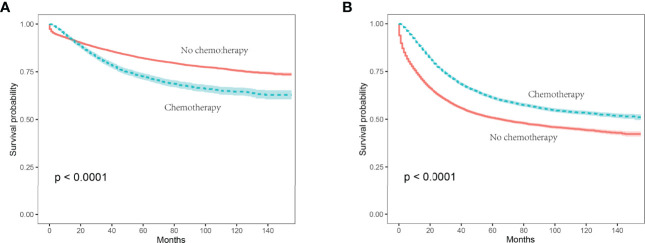
Kaplan-Meier survival curves were plotted to calculate the CSS rate, and comparisons of survival difference between chemotherapy and no chemotherapy subgroups were performed using the log-rank test. **(A)** In stage II colon cancer patients, the 5-year CSS rates of no chemotherapy and chemotherapy groups were 82.0% and 72.4%, respectively (P < 0.001); **(B)** in stage III colon cancer patients aged 70 years or older, the 5-year CSS rates of no chemotherapy and chemotherapy groups were 50.7% and 61.3%, respectively (P < 0.001).

In stage II colon cancer patients aged 70 years or older, the Kaplan-Meier survival analysis showed that the 5-year CSS rates of no chemotherapy and chemotherapy groups were 82.0% and 72.4%, respectively (P < 0.001), indicating a relatively poor survival of stage II colon cancer patients with the receipt of chemotherapy (**Figure 2A**).

Apart from this, we performed multivariate Cox proportional analysis of CSS for the prognostic characteristics in colon cancer patients. As shown in **Table 3**, for the whole cohort, race, tumor grade, primary tumor site, and the receipt of chemotherapy were independent prognostic determinants of CSS in stage II colon cancer patients aged 70 years or older. Using the white race as a reference, other races (HR = 1.137, 95% CI: 1.064-1.216, P < 0.001) had a poorer prognosis; using grade I/II as a reference, grade III/IV (HR = 1.178, 95% CI: 1.108-1.253, P < 0.001) had a poorer prognosis; using the right colon as a reference, the left colon (HR = 1.420, 95% CI: 1.349-1.495, P < 0.001) had a poorer prognosis. More importantly, patients with chemotherapy receipt were independently associated with a 44.6% higher cancer-specific mortality rate (HR = 1.446, 95% CI: 1.346-1.554, P < 0.001) compared with those who do not receive chemotherapy after adjusting for TNM stage, race, sex, year of diagnosis, tumor grade, primary tumor site, and histology.

**Table 3:** Multivariate Cox regression analyses for survival in stage II colon cancer.

Variables	N		Stepwise multivariate analysis	
			HR (95%CI)	P value
**Race**			<.001
**White**	30,170	1.0	
**Other**	4929	1.137 (1.064-1.216)	
**Sex**			0.520
**Male**	15,566	1.0	
**Female**	19,533	1.016 (0.967-1.068)	
**Year of diagnosis**			0.281
**2004-2008**	20,880	1.0	
**2009-2012**	14,219	0.972 (0.923-1.024)	
**Grade**			<.001
**I/II**	27,528	1.0	
**III/IV**	6886	1.178 (1.108-1.253)	
**Unknown**	685	1.385 (1.177-1.629)	
**Primary tumor site**			<.001
**Right colon**	24,398	1.0	
**Left colon**	10,701	1.420 (1.349-1.495)	
**Histology**			0.058
**Adenocarcinoma**	31,077	1.0	
**Mucinous adenocarcinoma/signet ring cell carcinoma**	4022	0.925 (0.853-1.003)	
**Chemotherapy**			<.001
**No**	32,046	1.0	
**Yes**	3053	1.446 (1.346-1.554)	

### The Effect of Adjuvant Chemotherapy in Stage III Colon Cancer Patients Aged 70 Years or Older

In stage III colon cancer patients aged 70 years or older, the Kaplan-Meier survival analysis showed that the 5-year CSS rates of no chemotherapy and chemotherapy groups were 50.7% and 61.3%, respectively (P < 0.001), indicating a relatively poor survival of stage III colon cancer patients without the receipt of chemotherapy (**Figure 2B**).

The results of multivariate Cox proportional analysis of CSS for the prognostic characteristics in colon cancer patients are shown in **Table 4**. For the whole cohort, race, year of diagnosis, tumor grade, histology, and the receipt of chemotherapy were independent prognostic determinants of CSS in stage III colon cancer patients aged 70 years or older. Using the white race as a reference, other races (HR = 1.089, 95% CI: 1.042-1.139, P < 0.001) had a poorer prognosis; using year of diagnosis between 2004 and 2008 as a reference, year of diagnosis between 2009 and 2012 (HR = 0.938, 95% CI: 0.906-0.971, P < 0.001) had a more favorable prognosis; using grade I/II as a reference, grade III/IV (HR = 1.435, 95% CI: 1.385-1.487, P < 0.001) had a poorer prognosis; using adenocarcinoma as a reference, mucinous adenocarcinoma/signet ring cell carcinoma (HR = 1.077, 95% CI: 1.023-1.133, P = 0.004) had a poorer prognosis. Furthermore, patients who received chemotherapy were independently associated with a 35.8% lower cancer-specific mortality rate (HR = 0.642, 95% CI: 0.620-0.665, P < 0.001) compared with those who did not receive chemotherapy after adjusting for TNM stage, race, sex, year of diagnosis, tumor grade, primary tumor site, and histology.

**Table 4:** Multivariate Cox regression analyses for survival in stage III colon cancer.

Variables	N		Stepwise multivariate analysis	
			HR (95%CI)	P value
**Race**			<.001
**White**	29,056	1.0	
**Other**	5791	1.089 (1.042-1.139)	
**Sex**			.866
**Male**	15,458	1.0	
**Female**	19,389	0.997 (0.964-1.032)	
**Year of diagnosis**			<.001
**2004-2008**	20,477	1.0	
**2009-2012**	14,370	0.938 (0.906-0.971)	
**Grade**			<.001
**I/II**	22,706	1.0	
**III/IV**	11,375	1.435 (1.385-1.487)	<.001
**Unknown**		1.231 (1.099-1.379)	<.001
**Primary tumor site**			.657
**Right colon**	24,242	1.0	
**Left colon**	10,605	0.992 (0.955-1.029)	
**Histology**			.004
**Adenocarcinoma**	30,631	1.0	
**Mucinous adenocarcinoma/signet ring cell carcinoma**	4216	1.077 (1.023-1.133)	
**Chemotherapy**			<.001
**No**	21,187	1.0	
**Yes**	13,660	0.642 (0.620-0.665)	

## Discussion

The risk of oncogenesis is highly associated with advanced age and the elderly occupy a sizable proportion of colon cancer patients. In clinical practice, physicians are more prone to bias that advanced age could not tolerate chemotherapy or that the survival benefit of adjuvant therapy is not worth the risks in these elderly patients (12). In fact, clinicians should rely more on an assessment of biological age rather than chronological age in making treatment decisions for colon cancer patients (13). In addition, older patients were more likely to experience surgical complications than younger patients and patient’s suboptimal postoperative recovery would also affect performance status, making these patients not willing to receive adjuvant chemotherapy (14). As a key factor affecting chemotherapy receipt, older age was associated with decreased receipt of chemotherapy (15, 16).

Surgical treatment is the most important treatment modality for colon cancer. However, it has recently been found that conventional colon surgery may not be sufficient when compared with standard surgery for rectal cancer (total mesorectal excision, TME) (17–19). Resection along the embryonic avascular mesorectal fascia plane could yield a complete specimen, including regional lymphatics, blood vessels, and surrounding adipose tissue bounded by the mesorectum (20). This could reduce the rate of local recurrence and improve disease-free and overall survival, and more research about this is needed in the future (21).

The first adjuvant chemotherapy regimen to demonstrate efficacy in colon cancer was the FULV regimen, which reduced the five-year risk of death by approximately 15% compared with surgery alone (22–24). Then in the MOSAIC study, the researchers found that the addition of oxaliplatin for 6 months (FOLFOX or CAPOX regimen) increased the efficacy of chemotherapy, which could increase the 6-year DFS and OS rates to 73.3% and 78.5%, respectively, as compared to 67.4% and 76% for fluoropyrimidine alone, and this result was confirmed in subsequent NSABPC-07 and XELOXA trials (11, 25–27).

However, the effectivity of adjuvant chemotherapy in elderly colon cancer patients was not clear, which was partially attributed to the problem that elderly colon cancer patients have been underrepresented or even excluded in clinical trial (28–31). It has been reported that only 35% of the patients > 65 years were asked by clinical investigators to participate in clinical trials, whereas 51% of patients < 65 years were asked for such trials (32). Hence, we conducted this retrospective analysis aiming to provide more evidence to guide adjuvant chemotherapy decisions in elderly colon cancer patients.

In this study, with 69,946 stage II/III patients aged 70 years or older identified, we conducted a retrospective analysis to assess the efficacy of adjuvant therapy in stage II/III colon cancer patients aged 70 years or older. First, Chi-square test of the baseline characteristics showed that stage III, other races, male, diagnosed between 2009 and 2012, grade III/IV, and left colon cancer were more likely to be associated with the receipt of adjuvant chemotherapy; compared with stage II patients, stage III colon cancer patients were more likely to be associated with the receipt of adjuvant chemotherapy than other races, grade III/IV, and mucinous adenocarcinoma/signet ring cell carcinoma. Then, the effect of adjuvant chemotherapy was assessed in stage II colon cancer patients aged 70 years or older. The Kaplan-Meier survival analysis indicated a relatively poor survival of stage II colon cancer patients with the receipt of chemotherapy and the 5-year CSS rates of no chemotherapy and chemotherapy groups were 82.0% and 72.4%, respectively (P < 0.001). Multivariate Cox proportional analysis indicated that race, tumor grade, primary tumor site, and the receipt of chemotherapy were independent prognostic determinants of CSS in stage II colon cancer patients aged 70 years or older. More importantly, patients with chemotherapy receipt were independently associated with a 44.6% lower cancer-specific mortality rate compared with those who do not receive chemotherapy after adjusting for TNM stage, race, sex, year of diagnosis, tumor grade, primary tumor site, and histology, which was in line with the result of the Kaplan-Meier survival analysis.

Finally, the effect of adjuvant chemotherapy was assessed in stage III colon cancer patients aged 70 years or older. The Kaplan-Meier survival analysis indicated a relatively poor survival of stage II colon cancer patients with the receipt of chemotherapy and the 5-year CSS rates of no chemotherapy and chemotherapy groups were 50.7% and 61.3%, respectively (P < 0.001). Multivariate Cox proportional analysis indicated race, year of diagnosis, tumor grade, histology, and the receipt of chemotherapy were independent prognostic determinants of CSS in stage III colon cancer patients aged 70 years or older. What is more, patients with chemotherapy receipt were independently associated with a 35.8% lower cancer-specific mortality rate compared with those who did not receive chemotherapy after adjusting for TNM stage, race, sex, year of diagnosis, tumor grade, primary tumor site, and histology.

In 2013, Kim et al. evaluated the efficacy of adjuvant chemotherapy in 382 elderly colon cancer patients and showed the survival benefit in stage III colon cancer patients offered by adjuvant chemotherapy though adjuvant chemotherapy was not effective in elderly patients with stage II colon cancer, which was consistent with our findings. Therefore, the present study further confirmed Kim et al.’s viewpoint that adjuvant chemotherapy should be considered for stage III colon cancer patients aged 70 years or older, but the chemotherapy benefit in elderly stage II colon cancer was not truly clear.

According to the clinical practice guidelines promulgated by the American Society of Clinical Oncology (ASCO), stage II colon cancer patients with high-risk factors (including T4, sampling of fewer than 12 lymph nodes in the surgical specimen, perineural or lymphovascular invasion, poorly or undifferentiated tumor grade, intestinal obstruction, tumor perforation, or grade BD3 tumor budding) should be offered adjuvant chemotherapy (33). Those stage II colon cancer patients without high-risk factors did not receive adjuvant chemotherapy and usually had a good prognosis, which might also be a cause of suboptimal chemotherapy benefit in elderly stage II colon cancer. However, our analysis did not address this aspect owing to the lack of information about high-risk factors, and this aspect should be explored in future studies.

Taken together, here we have conducted a retrospective study with a large sample size and provide more evidence for that adjuvant chemotherapy should be considered in the treatment for stage III colon cancer patients aged 70 years or older, but the chemotherapy benefit in elderly stage II colon cancer is suboptimal. Future prospective studies to validate our results are therefore highly warranted. Moreover, clinical researchers should support more elderly patients to participate in relevant randomized controlled clinical studies in the future.

We also acknowledge that there are flaws in this study. First, as mentioned above, our research does not address the aspect of whether high-risk factors were the cause of suboptimal chemotherapy benefit in elderly stage II colon cancer owing to the lack of information about high-risk factors. Second, comorbidities and performance status of patients which could influence their willingness to accept chemotherapy are not available because of the inherent limitations of the database. Finally, there are inherent drawbacks of the retrospective study design, prospective studies to validate our results are therefore highly warranted.

In conclusion, this work provides further evidence that adjuvant chemotherapy should be considered in the treatment for stage III colon cancer patients aged 70 years or older, but the chemotherapy benefit in elderly stage II colon cancer is suboptimal. Moreover, clinical researchers should support more elderly patients to participate in relevant randomized controlled clinical studies in the future.

## Data Availability Statement

Publicly available datasets were analyzed in this study. This data can be found here: The datasets analyzed in this study are collected from SEER repository (https://seer.cancer.gov/).

## Author Contributions

YX and CM conceived and designed the study; XC, JT, XX, and WG extracted and analyzed the data; XC, JT, XX, LQ, HQ, and ZJ participated in the interpretation of data, and wrote the manuscript; all authors read and approved the final manuscript.

## Conflict of Interest

The authors declare that the research was conducted in the absence of any commercial or financial relationships that could be construed as a potential conflict of interest.

## Publisher’s Note

All claims expressed in this article are solely those of the authors and do not necessarily represent those of their affiliated organizations, or those of the publisher, the editors and the reviewers. Any product that may be evaluated in this article, or claim that may be made by its manufacturer, is not guaranteed or endorsed by the publisher.
